# Mechanistic 5’UTR Variant Scoring Expands Rare Variant Discovery in the UK Biobank

**DOI:** 10.64898/2026.09.09.26362607

**Published:** 2026-09-11

**Authors:** Matthieu Chaldebas, Khoren Ponsin, Haralambos A. Mourelatos, Yoann Seeleuthner, Clément Conil, Jonathan Bohlen, Jean-Laurent Casanova, Peng Zhang, Aurélie Cobat

## Abstract

The 5′ untranslated region (5′UTR) regulates protein output through upstream open reading frames (uORFs) and Kozak context, yet most deleteriousness scores rely heavily on evolutionary conservation of its nucleotide positions. Using 5ULTRA, a machine-learning classifier trained on 5′UTR regulatory biology, we annotated rare and low-frequency 5′UTR variants in 408,423 UK Biobank participants. We tested gene-level associations for all 59 quantitative blood-count and serum biochemistry traits. We identified 58 genome-wide significant gene-phenotype associations and CADD 38, including 24 shared, 34 exclusive to 5ULTRA, and 14 to CADD. Removing 5ULTRA-annotated variants eliminated 15 of 38 CADD associations, indicating that a fraction of CADD’s performance depends on uORF and Kozak architecture. The 18 associations absent from a published UK Biobank 5′UTR study included 11 that were exclusive to 5ULTRA. Among these, *NELFCD* , a subunit of the RNA polymerase II pausing complex with no established role in megakaryopoiesis, reached −log₁₀ *P* = 50 for platelet distribution width. Associations were most often driven by variants predicted to suppress translation (13 of 16 directionally resolved associations; *P* = 0.021). Gene-level effects of 5′UTR repressor variants correlated with those of coding protein-truncating variants (r = 0.68, *P* = 3.7 × 10⁻⁴), placing them on the same phenotypic scale. Associations tested in non-European participants showed 89% directional concordance (r = 0.84), supporting shared regulatory effects across ancestries. Mechanistic 5′UTR annotation therefore recovers a translational layer of phenotypic variation that generic deleteriousness scores based on evolutionary constraints miss.

## Full Text Availability

The license terms selected by the author(s) for this preprint version do not permit archiving in PMC. The full text is available from the preprint server.

